# Objectifying Facial Expressivity Assessment of Parkinson's Patients: Preliminary Study

**DOI:** 10.1155/2014/427826

**Published:** 2014-11-13

**Authors:** Peng Wu, Isabel Gonzalez, Georgios Patsis, Dongmei Jiang, Hichem Sahli, Eric Kerckhofs, Marie Vandekerckhove

**Affiliations:** ^1^Department of Electronics and Informatics, Vrije Universiteit Brussel, 1050 Brussels, Belgium; ^2^Shaanxi Provincial Key Lab on Speech and Image Information Processing, Northwestern Polytechnical University, Xi'an, China; ^3^Department of Physical Therapy, Vrije Universiteit Brussel, 1050 Brussels, Belgium; ^4^Department of Experimental and Applied Psychology, Vrije Universiteit Brussel, 1050 Brussels, Belgium

## Abstract

Patients with Parkinson's disease (PD) can exhibit a reduction of spontaneous facial expression, designated as “facial masking,” a symptom in which facial muscles become rigid. To improve clinical assessment of facial expressivity of PD, this work attempts to quantify the dynamic facial expressivity (facial activity) of PD by automatically recognizing facial action units (AUs) and estimating their intensity. Spontaneous facial expressivity was assessed by comparing 7 PD patients with 8 control participants. To voluntarily produce spontaneous facial expressions that resemble those typically triggered by emotions, six emotions (amusement, sadness, anger, disgust, surprise, and fear) were elicited using movie clips. During the movie clips, physiological signals (facial electromyography (EMG) and electrocardiogram (ECG)) and frontal face video of the participants were recorded. The participants were asked to report on their emotional states throughout the experiment. We first examined the effectiveness of the emotion manipulation by evaluating the participant's self-reports. Disgust-induced emotions were significantly higher than the other emotions. Thus we focused on the analysis of the recorded data during watching disgust movie clips. The proposed facial expressivity assessment approach captured differences in facial expressivity between PD patients and controls. Also differences between PD patients with different progression of Parkinson's disease have been observed.

## 1. Introduction

One of the manifestations of Parkinson's disease (PD) is the gradual loss of facial mobility and “mask-like” appearance. Katsikitis and Pilowsky (1988) [1] stated that PD patients were rated as significantly less expressive than an aphasic and control group, on a task designed to assess spontaneous facial expression. In addition, the spontaneous smiles of PD patients are often perceived to be “unfelt,” because of the lack of accompanying cheek raises [2]. Jacobs et al. [3] confirmed that PD patients show reduced intensity of emotional facial expression compared to the controls. In order to assess facial expressivity, most research relies on subjective coding of the implied researchers, as in aforementioned studies. Tickle-Degnen and Lyons [4] found that decreased facial expressivity correlated with self-reports of PD patients as well as the Unified Parkinson's Disease Rating Scale (UPDRS) [5]. PD patients, who rated their ability to facially express emotions as severely affected, did demonstrate less facial expressivity.

In this paper, we investigate automatic measurements of facial expressivity from video recorded PD patients and control populations. To the best of our knowledge, in actual research, few attempts have been made for designing a computer-based quantitative analysis of facial expressivity of PD patient. To analyze whether Parkinson's disease affected voluntary expression of facial emotions, Bowers et al. [6] videotaped PD patients and healthy control participants while they made voluntary facial expression (happy, sad, fear, anger, disgust, and surprise). In their approach, the amount of facial movements change and timing have been quantified by estimating an* entropy score* plotted over time. The entropy is a measure of pixel intensity change that occurred over the face as it moved during expression. Also they computed the time it took an expression to reach its peak entropy value from the onset of each trial. Using both measures, entropy and time, the authors demonstrated that less movements occurred over the face of PD patients when they were asked to mimic a target expression relative to control.

Despite its good results, the above described amount of facial movements does not directly relate to measuring facial muscles activity. Indeed, facial expressions are generated by contractions of facial muscles, which lead to subtle changes in the area of the eyelids, eye brows, nose, lips, and skin texture, often revealed by wrinkles and bulges. To measure these subtle changes, Ekman and Friesen [7] developed the Facial Action Coding System (FACS). FACS is a human-observer-based system designed to detect subtle changes in facial features and describes facial expressions by action units (AUs). AUs are anatomically related to contraction of specific facial muscles. They can occur either singly or in combinations. Simons et al. in [2] used the FACS to analyze facial expressivity of PD patients versus control. In their study, odor was used as a means of inducing facial expression. Certified FACS coders annotated the facial expressions. A total facial activity (TFA) measure, estimated as the total number of displayed AUs, was used to assess facial expressions. The authors demonstrated that the TFA measure revealed that compared to controls, PD patients have reduced level of facial activity in reaction to unpleasant odors.

Estimating only the number of displayed AUs does not completely capture the dimensions of facial masking that are present in PD and defined in the* Interpersonal Communication Rating Protocol*-Parkinson's Disease Version (ICRP-IEB) [9]. The ICRP-IEB facial expressivity is based on the FACS. It defines expressivity in terms of (i)* frequency*, that is, how often a behavior or movement occurs, (ii)* duration*, that is, how long a behavior or movement lasts, and (iii)* intensity or degree* being the strength, force, or level/amount of emotion or movement.

In this study, we propose a system that (i) automatically detects faces in a video stream, (ii) codes each frame with respect to 11 action units, and (iii) estimates a facial expressivity as function of frequency, duration, and intensity of AUs. Although there is already a substantial literature on automatic expression and action unit recognition, it still remains an active area of study due to the challenging nature of the problem [10]. Moreover, in contrast to AU detection, there is scarce work in the literature on AU intensity estimation. The proposed facial expressivity quantity makes use of the output of a previously developed automatic AU recognition system [11] based on support vector machines (SVM) and AdaBoost. To determine the AU intensity, the resulting AU distance measure, from the AU SVM classifier, is mapped to the estimates of probability using the Platt scaling algorithm. Platt scaling [12] refers to a technique whereby a score-to-probability calibration curve is calculated using the training set. Frame-by-frame intensity measurements are then used to estimate facial expression dynamics which were previously intractable by human coding.

## 2. Methods

### 2.1. Pilot Study

Our study aims to quantify facial expressivity dynamics of PD patients. Thus, gathering usable qualitative emotional data is the essential step prior to the analysis of facial behaviors. To voluntarily produce spontaneous facial expressions that resemble those typically triggered by emotions, in our study, six emotions (amusement, sadness, anger, disgust, surprise, and fear) were elicited using movie clips. During the movie clips, physiological signals and frontal face video of the participants were recorded. Fifteen participants, 7 PD patients and 8 healthy control persons, participated in the pilot study. After each movie, the participants were asked to rate the intensity of dominant emotions they experienced while watching the movie clips. The evaluation of the participant's self-reports showed that the disgust-induced emotion was significantly higher than the other emotions. Thus we focused on the analysis of the recorded data during watching disgust movie clips.

### 2.2. Emotion Induction Protocol

Based on the studies of Gross and Levenson [13], Hagemann et al. [14], Lisetti and Nasoz [15], Hewig et al. [16], Westerink et al. [17], and Schaefer et al. [18], we composed a set of movie clips [19]. For each emotion (amusement, sadness, anger, disgust, surprise, fear, and neutral) two excerpts were used, as listed in Table 1. In the sequel, we will refer to move clips as *excerpt*#*i*; *i* = 1, 2. For example, the two surprise movie clips will be denoted as *surprise*#1 and *surprise*#2.

**Figure 1:**
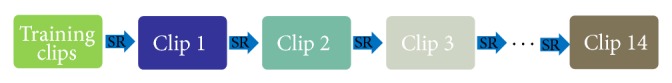
Emotion elicitation protocol (SR indicates self-report).

The used protocol is depicted in Figure 1. The participants were told to watch 2 training movie clips and the 14 movie clips of Table 1. The movie clips were shown randomly, 2 successive ones with different emotions. After each video clip the participants filled in a questionnaire (i.e., self-report) with their emotion-feeling (amusement, sadness, anger, disgust, surprise, fear, and neutral) and rated the strength of their responses using a 7-point scale.

**Figure 2:**
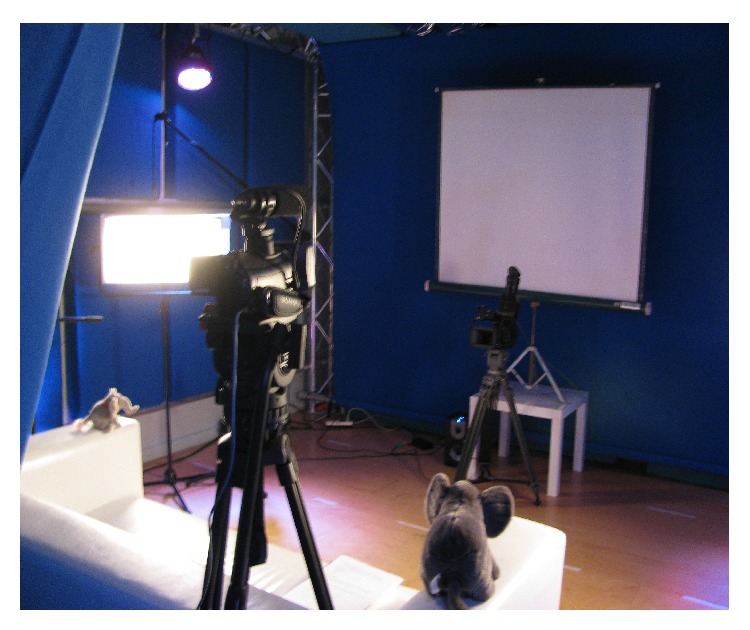
Experimental setup.

The data recording took place in a sound-isolated lab under standardized lighting condition. The movie clips were watched by the participants while sitting on a sofa that was facing a screen with dimensions 1.25 by 1.25 m (see Figure 2).

### 2.3. Participants

This pilot study considered seven PD patients (3 men and 4 women, between the ages of 47 and 76 years, durations of PD ranged from 1.5 to 13 years) and eight control participants (5 men and 3 women, between the age of 27 and 57 years). The control participants were recruited from the VUB ETRO department without specific characteristics. The PD patients were selected with the help of the Flemish Parkinson League. The study was approved by the committees on human research of the respective institutions and was completed in accordance with the Helsinki Declaration.

Based on the medical dossier as well as the feedback from the PD patients, we defined three PD categories. One patient had a very light form of PD and therefore was classified as the least severe case (denoted as LP). Another patient had the most severe form of PD of the whole group (MP). The rest of PD patients were situated between those two extremes; we denoted them by intermediate PD (IP).

### 2.4. Data Acquisition

During the movie clips, physiological signals and frontal face video of the participants were recorded. As physiological signal we recorded electromyography (EMG) and electrocardiogram (ECG). All the data channels (i.e., EMG, ECG, and videotape) were synchronized.

The ECG measures the activity of heart contractions. The physical action of the heart is induced by a local periodic electrical stimulation, and as a result a change in potential of 1.0-2.0 *μ*V is measured during a cardiac cycle between two surface electrodes [20]. Heart rate (HR) and heart rate variability (HRV) are the cardiovascular response features most often reported as indicators of emotions [21]. In this study, the ECG was measured at 512 Hz using a Shimmer.

**Figure 3:**
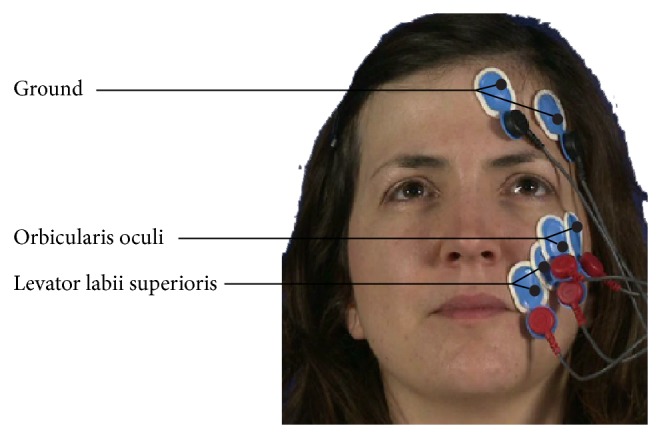
Locations of the EMG electrodes.

The EMG measures the frequency of muscle tension and contraction. Within each period of interest, the root mean square (RMS) and absolute mean value (AMV) are commonly used as features [22]. In this study, two facial muscles were measured, at 2000 Hz using the EMG Biomonitor ME6000, namely, the levator labii superioris (LLS) and the orbicularis oculi (OO), as illustrated in Figure 3.

The frontal face video of the participants was recorded for the purpose of quantifying facial expressivity. Following the (ICRP-IEB) [9], we selected 11 action units (AU1, AU2, AU4, AU6, AU7, AU9, AU12, AU20, AU23, AU25, and AU27) among the 41 ones defined by FACS.  Table 2 lists the used AUs, along with their associated facial muscles, as well as the ones defined by the measured EMG.

Our purpose, being the development of a nonobtrusive approach for facial expressivity assessment by automatically recognizing facial action units (AUs) and estimating their intensity, the ECG and EMG measurements have been included in our experimental protocol to quantitatively asses the emotional manipulation for inducing facial expressions and confirm facial muscle activity when expressing disgust expression. As our objective did not aim at physiologically investigating the effect of Parkinson's on facial EMG, only the LLS and OO were measured by the EMG as a complementary information to the considered AUs (see Table 2).

### 2.5. Data Preparation

It is an accepted practice to code a proportion of the observations or “thin-slices” of recorded data as a representation of the analyzed behavior [23]. In our experiments, 30 s data (ECG, EMG, and video record) extracts were selected, corresponding to the last 30 s segments of the shown video clips. Intuitively, one may think that longer segments of expressive behavior in persons with PD would give more information and thus increase the accuracy of analysis. However, Ambady and Rosenthal [24] found that the judgment performance did not significantly improve when using 5 minutes slices versus 30 s slices. This was also confirmed in [25, 26]. Note that baseline data were also selected from the stimuli neutral#1.

### 2.6. Physiological Data Processing

**Figure 4:**
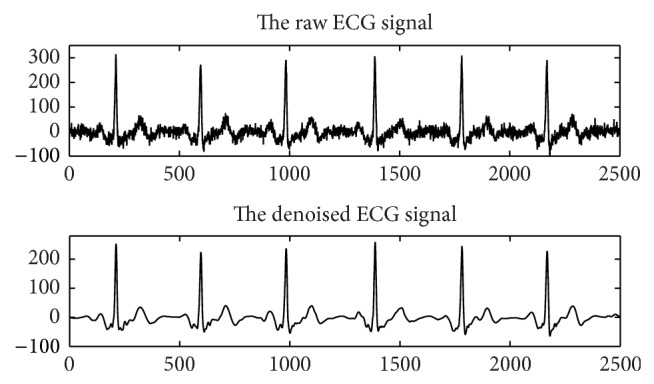
Example of physiological signal (ECG) denoising using the method proposed in [8].

Physiological signals need to be preprocessed prior to feature extraction in order to remove noise and enhance signal-to-noise ratio (SNR) [27, 28]. In this work, we make use of a newly developed signal denoising approach based on the empirical mode decomposition (EMD) [8]. Different from state-of-the-art methods [27–33], our approach estimates the noise level of each intrinsic mode functions (IMFs), rather than estimating the noise level of all IMFs using Donoho's strategy [34], prior to the reconstruction of the signal using the thresholded IMFs. Please refer to [8] for more details.  Figure 4 illustrates the denoising results.

#### 2.6.1. Electrocardiogram (ECG)

**Figure 5:**
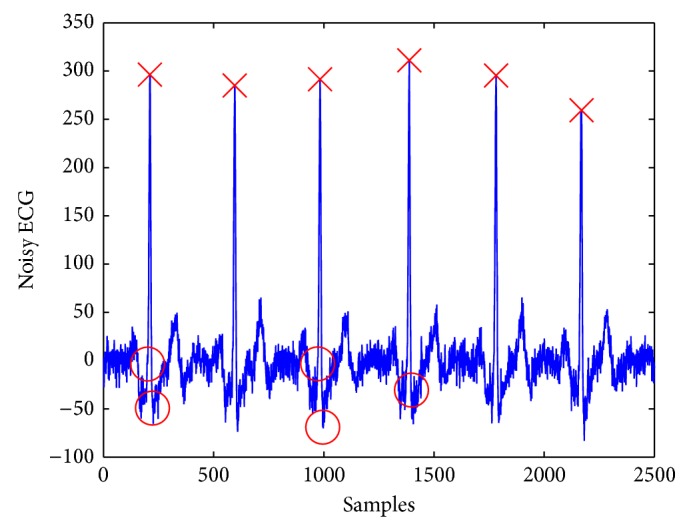
Change points detection.

Heart rate (HR) and heart rate variability (HRV) are the cardiovascular response features most often reported as indicators of emotion [21]. HR is computed using the time difference between two consecutive detected R peaks of the QRS complexes (i.e., RR interval) and is expressed in beats per minute. HRV is the variation of beat-to-beat HR. Thus, the first step in extracting HR and HRV starts from the exact detection of R peaks in the QRS complex. Thus the detection of QRS complex, in particular R peak detection, is the basis for ECG processing and analysis. Many approaches for R peaks detection have been proposed [35]. However, most of them are off-line and targeting the noiseless signal, which do not meet the requirements of many real-time applications. To overcome this problem, in [8], we proposed an approach based on a change point detection (CPD) algorithm for event detection in time series [36] that minimizes the error in fitting a predefined function using maximum likelihood. In our current implementation polynomial fitting functions of degree 1 have been selected empirically. An example of results is illustrated in Figure 5 which detects all R picks (cross) and some irrelevant change points (circle), which can be filtered out using a predetermined threshold. Once the R peaks were detected, HR and HRV features are estimated as the difference between the HR (HRV) estimated from the considered 30 s window (of the stimuli) and the one estimated from the neutral 30 s.

#### 2.6.2. Electromyogram (EMG)

The absolute mean value (AMV) is the most commonly used feature for identifying the strength of muscular contraction [22, 37], defined over the length, *N*, of the signal *x*(*t*) as follows:

(1) $$\begin{array}{c} \text{AMV}=\frac{1}{N}\overset{N}{\underset{t=1}{\sum }}\left|x\left(t\right)\right|. \end{array}$$

The AMV value during the considered 30 s window was expressed as a percentage of the mean amplitude during the 30 s neutral baseline. This percentage score was computed to standardize the widely different EMG amplitudes of individuals and thus to enable comparison between individuals and groups.

### 2.7. Design and Statistical Analysis

Preliminary analysis were performed to check the validity of the acquired data.

(*1) Manipulation Check*. We calculated the descriptive statistics (the mean and standard deviation) of self-reported emotional ratings to check if the participants' emotional experience was successfully manipulated. Then, Wilcoxon rank sum tests were performed to compare the self-report between groups (PD and control) and between the two clips of the same emotion.

(*2) Analysis of Physiological Parameters*. Physiological variables were tested for univariate significant differences, via repeated-measures analysis of variance (ANOVA), between groups (PD versus control) and between the disgust#1, disgust#2, and neutral#2 stimulants. Group and stimuli are the between-subjects and within-subject factors, respectively. Results were considered statistically significant at *P* < .05.

### 2.8. Facial Action Units Recognition

**Figure 6:**
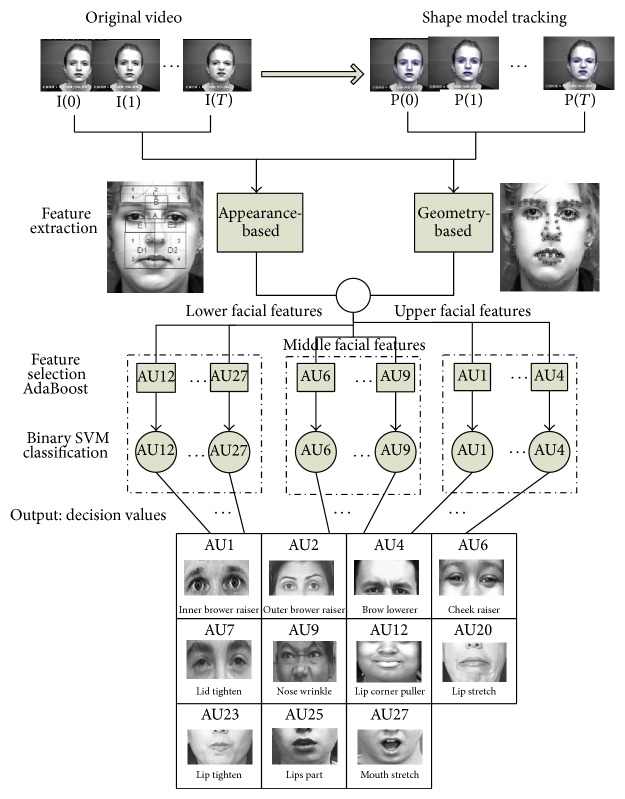
The AU recognition system.

In this study we make use of an automatic facial action units recognition system developed at our department [11]. This system allows context-independent recognition of the following action units: AU1, AU2, AU4, AU6, AU7, AU9, AU12, AU20, AU23, AU25, and AU27. The overall recognition scheme is depicted in Figure 6.

The head and facial features were tracked using the constrained shape tracking approach of [38]. This approach allows an automatic detection of the head and the tracking of a shape model composed of 83 landmarks. After the facial components have been tracked in each frame, both* geometry-based* features and* appearance-based* features are combined and fed to the AdaBoost (adaptive boosting) algorithm for feature selection. Finally, for each action unit, we used a binary support vector machine (SVM) for context-independent classification. Our system was trained and tested on the Kanade et al. DFAT-504 dataset [39]. The database consists of 486 sequences of facial displays that are produced by 98 university students from 18 to 30 years old, of which 65% is female. All sequences are annotated by certified FACS coders, start with a neutral face, and end with the apex of the expression. SVM [11] has been proven to be powerful and robust tools for AU classification.

A test sample (a new image) **z** can be classified according to the following decision function:

(2) $$\begin{array}{c} D\left(z\right)=\mathrm{sign}\left(h\left(z\right)\right). \end{array}$$

The output *h*(**z**) of a SVM is a distance measure between a test pattern and the separating hyperplane defined by the support vectors. The test sample is classified to the positive class (the AU to which it was trained) if *D*(**z**) = +1 and is classified to the negative class if *D*(**z**) = −1.

Unfortunately, we cannot use directly the output of a SVM as a probability. There is no clear relationship with the posterior class probability *P*(*y* = +1∣**z**) that the pattern **z** belongs to the class *y* = +1. Platt [12] proposed an estimate for this probability by fitting the SVM output *h*(**z**) with a sigmoid function as follows:

(3) $$\begin{array}{c} P\left(y=+1\;|\;z\right)=\frac{1}{1+\exp\left(Ah\left(z\right)+B\right)}. \end{array}$$

The parameters *A* and *B* are found using maximum likelihood estimation from the training set. The above equation has a mapping range in [0; 1].

### 2.9. Facial Expressivity

In this work we follow the recommendation of the* Interpersonal Communication Rating Protocol*-Parkinson's Disease Version (ICRP-IEB) [9], where the expressivity, based on the FACS, has been defined in terms of (i)* frequency*, that is, how often a behavior or movement occurs, (ii)* duration*, that is, how long a behavior or movement lasts, and (iii)* intensity or degree* being the strength, force, or level/amount of emotion or movement. In ICRP-IEB facial expressivity is coded according to the gestalt degree of intensity/duration/frequency of 7 types of facial expressive behavior (items), depicted in recorded videos of PDs, using a 5-point Likert type scale: 1 = low (with low to no movement or change or infrequent), 2 = fairly low, 3 = medium, 4 = fairly high, and 5 = high (very frequent, very active).  Table 3, lists 6 of the ICRP-IEB facial expressivity items and the corresponding AUs used in this study for estimating them. The last item is related to active mouth closure during speech, which was not used in our model, as the participants were not asked to speak during the experiments.

As our current AU recognition system considers only 11 AUs (AU1, AU2, AU4, AU6, AU7, AU9, AU12, AU20, AU23, AU25, and AU27), we did not consider blinking (AU45) in our facial expression formulation.

Following the criteria of the ICRP-IEB, we defined the facial expressivity of a participant as follows:

(4) $$\begin{array}{c} \text{EFE}={\text{AMC}}_{e}+{\text{AI}}_{e}-\left({\text{AMC}}_{n}+{\text{AI}}_{n}\right), \end{array}$$

where AMC_*e*_ and AMC_*n*_ are the amount of movement changes during the disgust and neutral facial expression, respectively. AI_*e*_ and AI_*n*_ are the intensity of the displayed AUs during the disgust and neutral facial expression, respectively.

**Figure 7:**
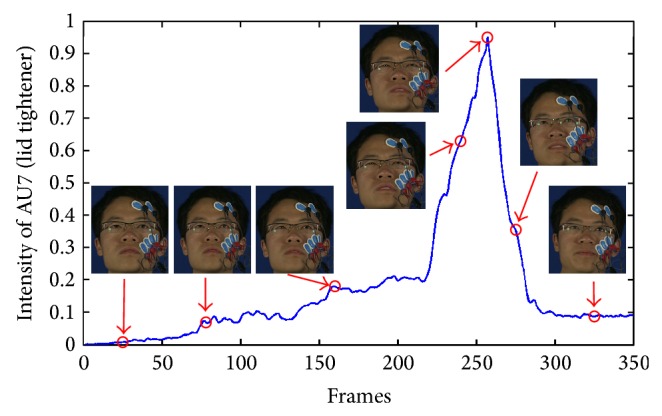
Example of AU intensity.

It has to be recalled that for all these estimations only the considered 30 s windows are used. The quantities AMC and AI refer to frequency and intensity, respectively. Their detailed formulation is given in the following sections. In order to assess the effectiveness of the proposed formulation we also compared it to the following definition of facial expression, where the baseline of neutral emotion was not considered. Consider the following:

(5) $$\begin{array}{c} \text{FE}={\text{AMC}}_{e}+{\text{AI}}_{e}. \end{array}$$

(*1) Intensity of Displayed AUs*. It has been shown in [40] that the output margin of the learned SVM classifiers contained information about expression intensity. Later Savran et al. [41] estimated the AU intensity levels, using logistic regression on SVM scores. In this work, we propose using Platt's probability, given by (3), as action unit intensity at frame *t*, *I*_*t*_(AU*i*) = *P*_AU*i*_^*t*^, with *P*_AU*i*_^*t*^ = *P*(*c* = AU*i*∣**z**_*t*_), is estimated using (3). The *I*_*t*_(AU*i*) time series is then smoothed using a Gaussian filter. We denote by ${\tilde{I}}_{t}(\text{AU}i)$ the smoothed value.  Figure 7 plots, for a participant, the smoothed intensity of the facial AU7, also illustrating its different temporal segments neutral, onset, apex, and offset.

Having defined the AU intensity, the intensity of displayed AUs during a voluntary facial expression (disgust or neutral) is given by

(6) $$\begin{array}{c} \text{AI}=\underset{i\in \text{DAUs}}{\sum }\frac{1}{N_{i}^{\prime }}\underset{t\in T_{i}}{\sum }{\tilde{I}}_{t}\left(\text{AU}i\right), \end{array}$$

where DAUs is the set of displayed (recognized) facial action units during the considered 30 s window, *T*_*i*_ is the set of frames where AU*i* is active, and *N*_*i*_′ is the cardinal of *T*_*i*_, being the number of frames where AU*i* is active.

(*2) Amount of Movement Change*. The amount of movement change during a voluntary facial expression (disgust or neutral) is given by

(7) $$\begin{array}{c} \text{AMC}=\underset{i\in \text{DAUs}}{\sum }\frac{1}{N_{i}^{\prime }}\underset{t\in T_{i}}{\sum }\left|{\tilde{I}}_{t}\left(\text{AU}i\right)-{\tilde{I}}_{t+1}\left(\text{AU}i\right)\right| \end{array}$$

with DAUs, *N*_*i*_′, and *T*_*i*_ as defined above.

## 3. Results and Discussion

### 3.1. Manipulation Check

Participants' emotion was successfully elicited using the movie clips of disgust. For both disgust#1 and disgust#2, most participants (14/15) self-reported the target emotion as their dominated emotion. For the other video clips the reported emotions are as follows: amusement#1 (9/15), amusement#2 (12/15), surprise#1 (11/15), surprise#2 (2/15), anger#1 (6/15), anger#2 (9/15), fear#1 (10/15), fear#2 (11/15), neutral#1 (13/15), and neutral#2 (13/15). Annotation of the video records, using the Anvil annotation tool [42], further confirmed that the movie clips of disgust induced the most reliable emotional data. Therefore, for further analysis, we decided to use only the data recorded during watching the disgust movie clips.

The Wilcoxon rank sum tests were implemented on the 14 disgusting self-reports. Results showed that there was no significant difference in self-reported emotional ratings between disgust#1 (*M* = 6.50, SD = .76) and disgust#2 (*M* = 6.64, SD = .63) and between PD group (*M* = 6.51, SD = .65) and control group (*M* = 6.64, SD = .74). This is what we expected since Vicente et al. [43] also reported that PD patients at different stages of the disease did not significantly differ from the controls in the self-reported emotional experience to presented movie clips.

### 3.2. Univariate Analysis of Group Effects

The EMG data from 3 participants (1 control and 2 PD) and the ECG data from 10 participants (4 control and 6 PD) were discarded because of not being well recorded due to sensor malfunctions. Results of the repeated-measures ANOVAs on single physiological variables showed that significant main effect of group on the LLS activity (*F*(1,12) = 3.38, *P* = .09), the OO activity (*F*(1,12) = 1.92, *P* = .19), HR (*F*(1,3) = .93, *P* = .41), and HRV (*F*(1,3) = .53, *P* = .83) was not found.  Table 4 shows the descriptive data for control and PD groups during exposure to the three movie clips disgust#1, disgust#2, and neutral#2 and the tests of significance comparison between groups using Wilcoxon rank sum tests. The Wilcoxon rank sum tests revealed that

1. comparable levels of baseline activity in control and PD groups over both the LLS and the OO were found;
2. although disgust#1 elicited more muscle activity over the LLS and the OO for control group than for PD group, this difference did not reach statistical significance;
3. disgust#2 elicited significantly more LLS activity for control than for PD.

These results indicated that PD displayed less muscle activity over the LLS when expressing disgust than control. In addition, disgust#2 induced more muscle activity over the LLS and the OO than disgust#1, which is consistent with the self-report and may be due to the fact that disgust#2 is slightly more disgusting than disgust#1.

### 3.3. Univariate Analysis of Stimuli Effects

For LLS, we found an effect of the stimuli on muscle activity (*F*(2,24) = 9.47, *P* = .001). Post-hoc tests indicated significant differences between disgust#1 and neutral#2 (*P* = .01) and between disgust#2 and neutral#2 (*P* = .01). Both disgust#1 (*M* = 2.82, SD = 1.92) and disgust#2 (*M* = 5.37, SD = 5.43) elicited more muscle activity than neutral#2 (*M* = 1.10, SD = .38). This main effect was qualified by a stimuli × group interaction (*F*(2,24) = 4.17, *P* = .028), which was consistent with the results of the Wilcoxon rank sum tests which compared the physiological responses between groups.

For OO, we also found an effect of the stimuli on muscle activity (*F*(2,24) = 5.45, *P* = .012). Post-hoc tests indicated significant difference only between disgust#1 and neutral#2 (*P* = .002). The disgust#1 (*M* = 2.32, SD = 1.57) elicited more muscle activity than neutral#2 (*M* = 1.35, SD = 1.11). No significant stimuli × group interaction effect (*F*(2,24) = 1.77, *P* = .192) was found.

We expected that the disgust clips elicited significantly more muscle activity over LLS than the neutral clip, because normally LLS is involved in producing the disgust facial expression [44]. The fact that OO was also significantly different was probably due to the following:

1. crosstalk [45], the LLS, and the OO lie in the vicinity of each other;
2. the disgust#1 elicited is not only a disgust but also a bit of amusement by the funny motion of the character and the background music so that the OO was also involved in producing the facial expression of amusement.

Note that disgust#2 (*M* = 3.64, SD = 4.32) elicited more muscle activity over OO (because of crosstalk) than neutral#2 (*M* = 1.14, SD = .87); however, unlike disgust#1, the difference did not reach statistical significance (because disgust#2 did not elicit amusement at all) which can also be interpreted.

Moreover, the main effect of stimuli on cardiac parameters for both HR (*F*(2,6) = .37, *P* = .70) and HRV (*F*(2,6) = 0.84, *P* = .48) was not found, which is not consistent with what we expected: unchanged [46, 47] or increased [48, 49] HR and increased HRV [21, 47]. This may be due to the fact that we did not have enough recorded ECG data for a statistical analysis.

### 3.4. Qualitative Analysis of Facial Expressivity

To qualitatively analyze facial expressivity we used the total facial activity TFA measure of [2, 50], being the total number of displayed AUs in response to the stimuli. Compared to the control (C), the PD groups (LP, IP, and MP) showed the attenuation of their facial activities (variable TFA) while watching disgusting movie clips (see Table 5). However, comparable TFA was found while watching neutral movie clips.

**Figure 8:**
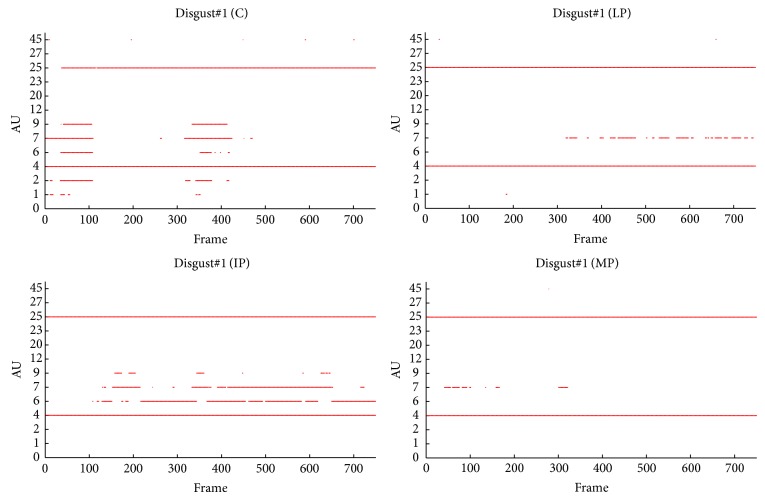
The facial activities while watching disgust#1.

**Figure 9:**
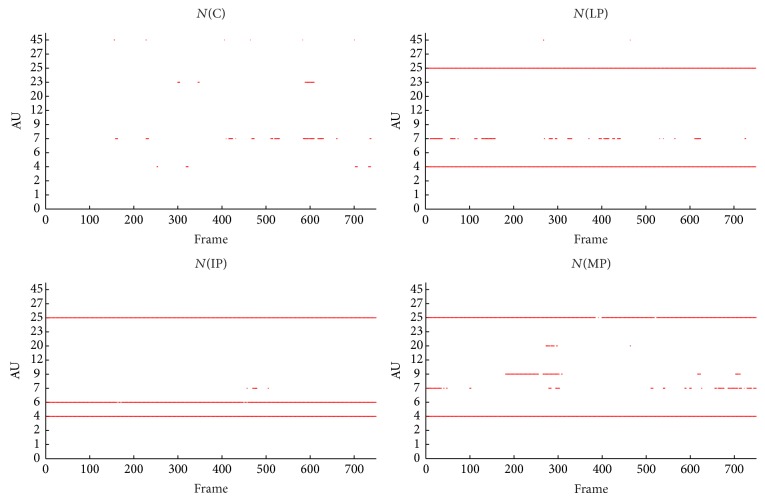
The facial activities while watching neutral#2.

Visual inspection of the of the displayed AUs, as illustrated in Figure 8, shows that AU1, AU2, AU6, AU9, and AU45 occurred more frequently for C, except for IP, who produced more frequently AU6 than C. A possible explanation is that the IP cannot deactivate AU6 even during neutral state. As shown in Figure 9, during neutral state, PD patients produced more continuous active action units, such as AU4 and AU25 (for all PD), AU6 (for IP), and AU9 (for MP). Moreover, certain AU combinations were considered likely to signify “disgust” on the basis of Ekman and Friesen's description of emotions [7]. “Disgust” AUs were considered as the combination of eyebrows lowerer (AU4), cheek raiser (AU6), and nose wrinkler (AU9). Because cheek raiser is very difficult to produce on demand without including other AUs, especially the lid tightener (AU7) [51], lid tightener was also taken into consideration; that is, the expected AU pattern of “disgust” was the combination of AU4, AU6, AU7, and AU9. Consistent with what we expected, C displayed 98 and 87 “disgust” frames while watching disgust#1 and disgust#2, respectively. The PD goup (LP, IP, and MP) did not display any “disgust” frames, only for IP who had 4 “disgust” frames while watching disgust#1. Furthermore, the IP displayed quite few frames with combinations of cheek raiser and lid tightener during neutral state. Instead, the cheek raiser alone was displayed mostly (see Figure 9), which indicates that IP patients have control problems for facial muscles combinations.

Analyzing the defined quantities AMC, AI, and FE using the segments with active AUs, as it can be seen from Table 5, the control C had higher facial expressivity than LP, IP, and MP during the disgusting state while MP performed the lowest facial expressivity. More specifically, compared to C, PD (LP, IP, and MP) had smaller values of variables AMC, AI, and FE while watching disgusting movie clips, especially for brow lower (AU4), nose wrinkler (AU9), and blink (AU45). C tended to show similar intensities of brow lower (variable *e*) with bigger variance (variables AMC and FE). In addition, C showed higher intensities of nose wrinkler with larger variance.

### 3.5. Quantitative Analysis of Facial Expressivity

**Figure 10:**
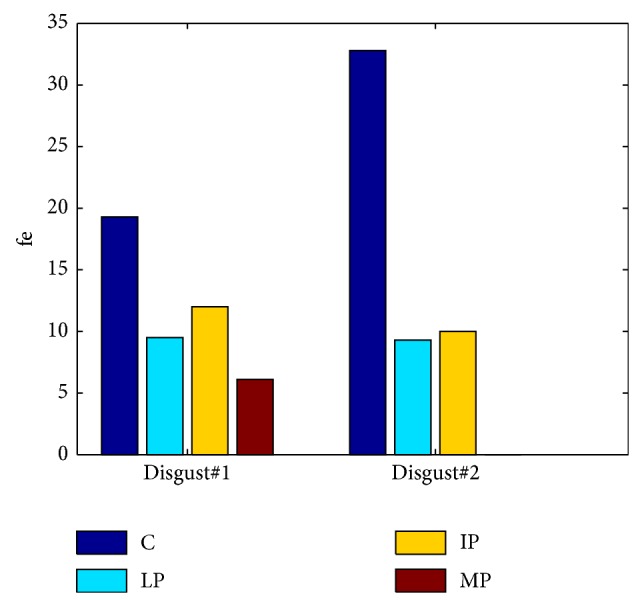
The quantified facial expressivity.

**Figure 11:**
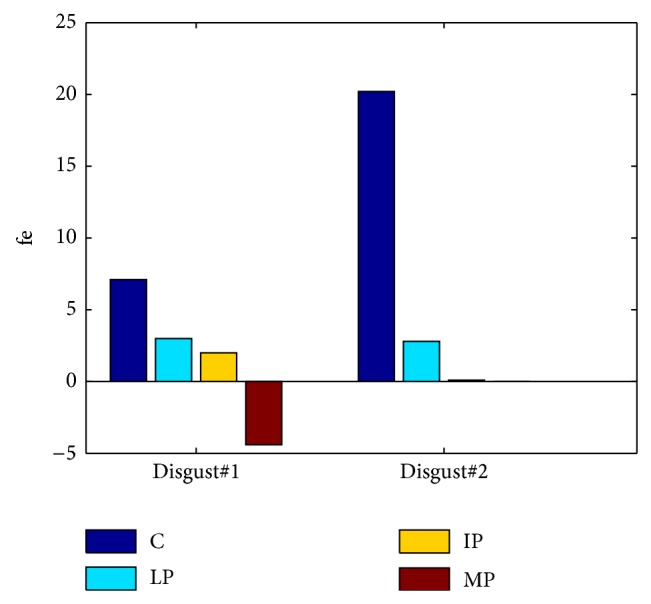
The quantified facial expressivity based on the improved function.

Figure 10 depicts the values of the facial expressivity equation (5) for the participants. We did not assess the facial expressivity of the MP patient during disgust#2, as he had his hand on front of his face during the experiments. As it can be seen, a significant difference between C and PD patients is present. C got the highest score while the lowest score was obtained by the MP patient. However, the score of the IP patient was slightly higher than the LP one, which is due to the fact that the LP and IP expressed “facial masking” in different ways: the LP patient showed the attenuation of the intensities of facial movements. On the contrary, the IP patient produced high intensities of facial movements not only in his emotional state but also during neutral state; that is, he cannot relax the muscles well. In order to take both kinds of “facial masking” into account, we computed the facial expressivity using (4). The results are shown in Figure 11. As it can be seen, the proposed facial expressivity allows distinguishing between control and PD patients. Moreover the facial expressivity decreases along with the increase of PD severity.

## 4. Conclusions

This study investigated the phenomenon of facial masking in Parkinson's patients. We designed an automated and objective method to assess the facial expressivity of PD patients. The proposed approach follows the methodology of the* Interpersonal Communication Rating Protocol*-Parkinson's Disease Version (ICRP-IEB) [9]. In this study, based on the Facial Action Coding System (FACS), we proposed a methodology that (i) automatically detects faces in a video stream, (ii) codes each frame with respect to 11 action units, and (iii) estimates a facial expressivity as function of* frequency*, that is, how often AUs occur,* duration*, that is, how long AUs last, and* intensity* being the strength of AUs.

Although, the proposed facial expressivity assessment approach has been evaluated in a limited number of subjects, it allows capturing differences in facial expressivity between control participants and Parkinson's patients. Moreover, facial expressivity differences between PD patients with different progression of Parkinson's disease have been assessed. The proposed method can capture these differences and give a more accurate assessment of facial expressivity for Parkinson's patients than what traditional observer based ratings allow for. This confirms that our approach can be used for clinical assessment of facial expressivity in PD. Indeed, nonverbal signals contribute significantly to interpersonal communication. Facial expressivity, a major source of nonverbal information, is compromised in Parkinson's disease. The resulting disconnect between subjective feeling and objective facial affect can lead people to form negative and inaccurate impressions of people with PD with respect to their personality, feelings, and intelligence. Assessing in an objective way the facial expressivity limitation of PD would allow developing personalized therapy to benefit facial expressivity in PD. Indeed Parkinson's is a progressive disease, which means that the symptoms will get worse as time goes on. Using the proposed assessment approach would allow regular facial expressivity assessment by therapist and clinicians to explore treatment options.

Future work will (i) improve the AU recognition system and extend it to more AUs and (ii) consider clinical usage of the proposed approach in a statistically significant PD patients population.

## Figures and Tables

**Table 1:** The selected movie clips listed with their sources.

Emotion	Excerpt's source	
	#1	#2
Amusement	Benny and Joone	The god father
Sadness	An officer and a gentleman	Up
Surprise	Capricorn one	Sea of love
Anger	Witness	Gandhi
Disgust	Pink flamingos	Trainspotting
Fear	Silence of the lambs	The shining
Neutral	Colour bar patterns	Hannah and her sisters

**Table 2:** FACS AUs and related muscles.

AU	FACS name	Facial muscle	Videotape	EMG
AU1	Inner brow raiser	Frontalis (pars medialis)	X	—
AU2	Outer brow raiser	Frontalis (pars lateralis)	X	—
AU4	Brow lowerer	Depressor glabellae, depressor supercilii, and CS	X	—
AU6	Cheek raiser	OO (pars orbitalis)	X	X
AU7	Lid tightener	OO (pars palpebralis)	X	X
AU9	Nose wrinkler	LLSAN	X	—
AU10	Upper lip raiser	LLS, caput infraorbitalis	—	X
AU12	Lip corner puller	Zygomaticus Major	X	—
AU20	Lip stretcher	Risorius	X	—
AU23	Lip tightener	Orbicularis Oris	X	—
AU25	Lips part	Depressor labii inferioris	X	—
AU27	Mouth stretch	Pterygoids, digastric	X	—
AU45	Blink	Contraction OO	—	X
AU46	Wink	OO	—	X

**Table 3:** Facial expressivity items defined in ICRP-IEB [9] and used AUs.

Item	Gestalt degree	Related AUs
(1) Active expressivity in face	Intensity	11 AUs
(2) Eyebrows raising	Intensity + frequency	AU1 and AU2
(3) Eyebrows pulling together	Intensity + frequency	AU4
(4) Blinking	Frequency	AU45
(5) Cheek raising	Intensity + frequency	AU6
(6) Lip corner puller	Intensity + frequency	AU12

**Table 4:** Statistical summary of physiological measures.

Variable	Stimuli	Control		PD		Sig.
		Mean	SD	Mean	SD	
LLS	Neutral#2	1.04	.34	1.16	.43	.71
	Disgust#1	3.36	2.04	2.27	1.77	.26
	Disgust#2	8.04	6.49	2.71	2.25	.04^*^

OO	Neutral#2	1.35	1.11	.94	.54	.81
	Disgust#1	2.82	1.77	1.81	1.28	.32
	Disgust#2	5.20	5.71	2.07	1.48	.13

HR	Neutral#2	1.72	2.77	2.76	—	—
	Disgust#1	−.53	2.64	5.12	—	—
	Disgust#2	2.22	5.53	5.65	—	—

HRV	Neutral#2	1.53	12.96	−7.65	—	—
	Disgust#1	8.01	11.45	26.34	—	—
	Disgust#2	14.86	35.64	14.92	—	—

^*^The two groups are significantly different; that is, *P* < .05.

^^“—” We did not compare the cardiac responses (i.e., HR and HRV) between groups, because due to technical failures we lost the ECG data for 10 participants and thus only 5 participants (4 controls and 1 PD) completed the ECG recording.

**Table 5:** Facial expressivity assessment based on different methods.

Var.	C^*^			LP^*^			IP^*^			MP^*^		
	*D*#1^*^	*D*#2^*^	*N*#2^*^	*D*#1^*^	*D*#2^*^	*N*#2^*^	*D*#1^*^	*D*#2^*^	*N*#2^*^	*D*#1^*^	*D*#2^*^	*N*#2^*^
TFA	8	8	4	5	5	4	5	6	4	4	—	5
AMC^◊^	13.9	27.7	10.8	6.7	6.5	4.3	8.2	6.2	6.9	3.9	—	8.2
AI	5.4	5.1	1.8	2.8	2.8	2.2	3.8	3.9	3.1	2.2	—	3.3
FE^◊^	72.2	75.1	24.6	20.9	18.7	14.2	44.4	35.6	26.2	13.5	—	32.2

^*^ *D*#1, *D*#2, *N*#2, C, LP, IP, and MP denote disgust#1, disgust#2, neutral#2, the control, the least, intermediate, and most severely form of Parkinson's patients, respectively.

^◊^presented in percentages.

“—” The face of the MP while watching *D*#2 was blocked by his hand.
